# 293. Lung Cancer and Hematologic Malignancy ( HM) Patients Are Associated with the Highest Risk of Progressing to Severe Disease and Mortality in Cancer Patients with COVID-19

**DOI:** 10.1093/ofid/ofab466.495

**Published:** 2021-12-04

**Authors:** Anne-Marie Chaftari, Alexandre Malek, Hiba Dagher, Ying Jiang, Arnaud Bayle, Arvinder Bhinder, Ana Fernandez Cruz, Bilal Siddiqui, Robert Somer, Tarcila Datoguia, Monica Slavin, Tomislav Dragvich, Edward Gorak, Nobuyoshi Mori, Ray Y Hachem, Issam I Raad

**Affiliations:** 1 The University of Texas MD Anderson Cancer Center, Houston, Texas; 2 UT MD Anderson Cancer Center, Houston, Texas; 3 MD Anderson Cancer Center, Houston, TX; 4 Gustave Roussy Cancer Hospital, Villejuif, Lorraine, France; 5 OhioHealth Physician Group, Marion, Ohio; 6 Unidad de Enfermedades Infecciosas, Madrid, Madrid, Spain; 7 Communty Health Network, Indiannapolis, Indiana; 8 Cooper University Healthcare, Camden, New Jersey; 9 Hospital Israelita Albert Einstein, Sao Paulo, Sao Paulo, Brazil; 10 National Centre for Infections in Cancer, Peter MacCallum Cancer Centre, Melbourne, Victoria, Australia; 11 Banner MD Anderson Cancer, Gilbert, Arizona; 12 Baptist MD Anderson Cancer Center, Jacksonville, Florida; 13 St. Luke’s International Hospital, Tokyo, Tokyo, Japan

## Abstract

**Background:**

Several studies have shown that underlying cancer is a risk factor for progression of COVID-19 to severe illness and fatal outcome but there is very little data that specifies which underlying cancer puts this patient population at the highest risk.

**Methods:**

We retrospectively collected de-identified data on 1115 cancer patients diagnosed with COVID-19 between January and November 2020, at 12 centers in Asia, Australia, Europe, North America, and South America. Patient characteristics including age, type of malignancy (hematologic malignancy [HM], lung cancer, and non-lung cancer were determined in association with severe illness as well as all-cause mortality within 30 days after COVID-19 diagnosis.

**Results:**

By multivariable logistic regression analysis, independent risk factors for 30-day mortality in cancer patients included age > 65 (OR 6.64; 95% CI 3.351to 12.55; p< 0.0001), ALC < 0.5 K/microliter (OR 2.10; 95% CI 1.16 to 3.79; p=0.014), and anemia at < 10g/dl (OR 2.41; 95% CI 1.30 to 4.44; p=0.005). Among cancer patients, the 30-day mortality rate was significantly higher in patients with lung cancer than in patients with non-lung cancer solid tumors, including those with lung metastases (22% vs 9%; p=0.001). Patients with HM tended to have higher 30-day mortality than patients with non-lung cancer solid tumors (13% vs 9% p=0.07) and tended to have a lower mortality rate than patients with lung cancer (p=0.07). Furthermore, HM patients were more likely to be lymphopenic and anemic at diagnosis as well as progress to LRTI and be placed on ventilatory support compared to non-lung cancer solid tumor patients ( p= or < 0.01). In addition, lung cancer and HM patients were more likely to develop hypoxia and require hospital admission than non-lung cancer solid tumor patients ( p=0.01).

**Conclusion:**

Lung cancer and HM patients are associated with the highest risk of progressing to severe disease and mortality in cancer patients with COVID-19. Hence, cancer patient population should be given the highest priority as far as prevention [vaccination with boosters if needed] as well as preemptive early therapy with monoclonal antibodies right after the onset of COVID-19.

**Disclosures:**

**Monica Slavin, MBBS,MD**, **F2G** (Advisor or Review Panel member)**Merck** (Advisor or Review Panel member)**Pfizer** (Advisor or Review Panel member)

